# The transmembrane peptide DWORF activates SERCA2a *via* dual mechanisms

**DOI:** 10.1016/j.jbc.2021.100412

**Published:** 2021-02-11

**Authors:** Ang Li, Samantha L. Yuen, Daniel R. Stroik, Evan Kleinboehl, Razvan L. Cornea, David D. Thomas

**Affiliations:** Department of Biochemistry, Molecular Biology, and Biophysics, University of Minnesota, Minneapolis, Minnesota, USA

**Keywords:** calcium ATPase, cardiac muscle, fluorescence resonance energy transfer, FRET, biosensor, DWORF, dwarf open reading frame, FACS, fluorescence-activated cell sorting, FLT, fluorescence lifetime, FRET, fluorescence resonance energy transfer, GFP, green fluorescent protein, HF, heart failure, PLB, protein phospholamban, RFP, red fluorescent protein, SERCA, sarcoplasmic reticulum calcium ATPase, SR, sarcoplasmic reticulum, TM, transmembrane, UMGC, University of Minnesota Genomics Center, WT, wild-type

## Abstract

The Ca-ATPase isoform 2a (SERCA2a) pumps cytosolic Ca^2+^ into the sarcoplasmic reticulum (SR) of cardiac myocytes, enabling muscle relaxation during diastole. Abnormally high cytosolic [Ca^2+^] is a central factor in heart failure, suggesting that augmentation of SERCA2a Ca^2+^ transport activity could be a promising therapeutic approach. SERCA2a is inhibited by the protein phospholamban (PLB), and a novel transmembrane peptide, dwarf open reading frame (DWORF), is proposed to enhance SR Ca^2+^ uptake and myocyte contractility by displacing PLB from binding to SERCA2a. However, establishing DWORF’s precise physiological role requires further investigation. In the present study, we developed cell-based FRET biosensor systems that can report on protein–protein interactions and structural changes in SERCA2a complexes with PLB and/or DWORF. To test the hypothesis that DWORF competes with PLB to occupy the SERCA2a-binding site, we transiently transfected DWORF into a stable HEK cell line expressing SERCA2a labeled with a FRET donor and PLB labeled with a FRET acceptor. We observed a significant decrease in FRET efficiency, consistent with a decrease in the fraction of SERCA2a bound to PLB. Surprisingly, we also found that DWORF also activates SERCA’s enzymatic activity directly in the absence of PLB at subsaturating calcium levels. Using site-directed mutagenesis, we generated DWORF variants that do not activate SERCA, thus identifying residues P15 and W22 as necessary for functional SERCA2a–DWORF interactions. This work advances our mechanistic understanding of the regulation of SERCA2a by small transmembrane proteins and sets the stage for future therapeutic development in heart failure research.

Sarcoplasmic reticulum (SR) calcium ATPase (SERCA) plays a major role in regulating cytosolic Ca^2+^ levels within muscle cells (1), by pumping Ca^2+^ from the cardiomyocyte sarcoplasm into the SR lumen to enable muscle relaxation (diastole) after each muscle contraction (systole) (2), using energy derived from ATP hydrolysis (3). There are three major domains on the cytoplasmic face of SERCA. The phosphorylation (P) and nucleotide-binding (N) domains form the catalytic site, while the actuator (A) domain is involved in the transduction of conformational changes required for the Ca-uptake function involving the transmembrane (TM) domain (4). Of the 12 known SERCA isoforms expressed in muscle and nonmuscle cells (5), SERCA2a is the principal isoform expressed in cardiac myocytes. Reduced SERCA2a expression or activity has been identified as a causative factor contributing to the development of heart failure (HF) in humans (6). Specifically, elevated cytoplasmic Ca^2+^ levels in cardiomyocytes cause impaired muscle relaxation during diastole and extended duration of systole, and these effects are strongly correlated with the pathophysiology of HF (7, 8), which is a major health concern worldwide (9). Therefore, activation of SERCA2a represents an attractive target to mitigate cardiomyocyte deficiencies associated with HF (10).

SERCA2a is regulated by several homologous single-pass, helical TM proteins, including phospholamban (PLB) (11, 12), a 52-residue protein that is coexpressed with SERCA2a in ventricular myocytes. Unphosphorylated PLB inhibits SERCA2a, but inhibition is relieved by either micromolar Ca^2+^ (systolic) or β-adrenergic stimulation of PLB phosphorylation (13). Hereditary mutations in PLB increase inhibitory potency and are associated with human HF (11). Therefore, gene and small-molecule therapies to increase SERCA2a activity, either by activating SERCA2a directly or by relieving inhibition by PLB, are of strong interest to the research and medical communities (11, 14, 15). One gene therapy approach has been to express noninhibitory PLB mutants, evaluated by their ability to displace the inhibitory wild-type (WT) PLB, as measured by fluorescence resonance energy transfer (FRET) between a donor fluorophore on SERCA2a and an acceptor fluorophore on PLB (16, 17, 18).

All long-known endogenous membrane protein regulators of SERCA (*e.g.*, PLB, sarcolipin) are inhibitory, but a SERCA-activating peptide termed dwarf open reading frame (DWORF) has recently been identified (19). DWORF activates SERCA2a in the SR of cardiac myocytes, where PLB is also present; leading to the hypothesis that DWORF activates SERCA2a indirectly by displacing PLB but has no direct effect on SERCA2a function (19). In the present study, we have investigated this mechanism by using fluorescence lifetime (FLT) detection of FRET, to determine quantitatively the interactions of PLB and DWORF (separately and in combination) with SERCA2a and correlate with functional assays of SERCA activity on the same samples (10, 17). With this approach, we find that DWORF increases the Ca-ATPase activity of SERCA not only by displacing the inhibitory PLB, but also through direct interactions with SERCA2a in the absence of PLB. Using site-directed mutagenesis, we identified two DWORF residues, P15 and W22 (in the human isoform), as essential for activation of SERCA2a. These findings provide key insights needed to develop effective therapies for heart failure.

## Results

### Expression, localization, and FRET measurements of SERCA2a–DWORF biosensor

**Figure 1:**
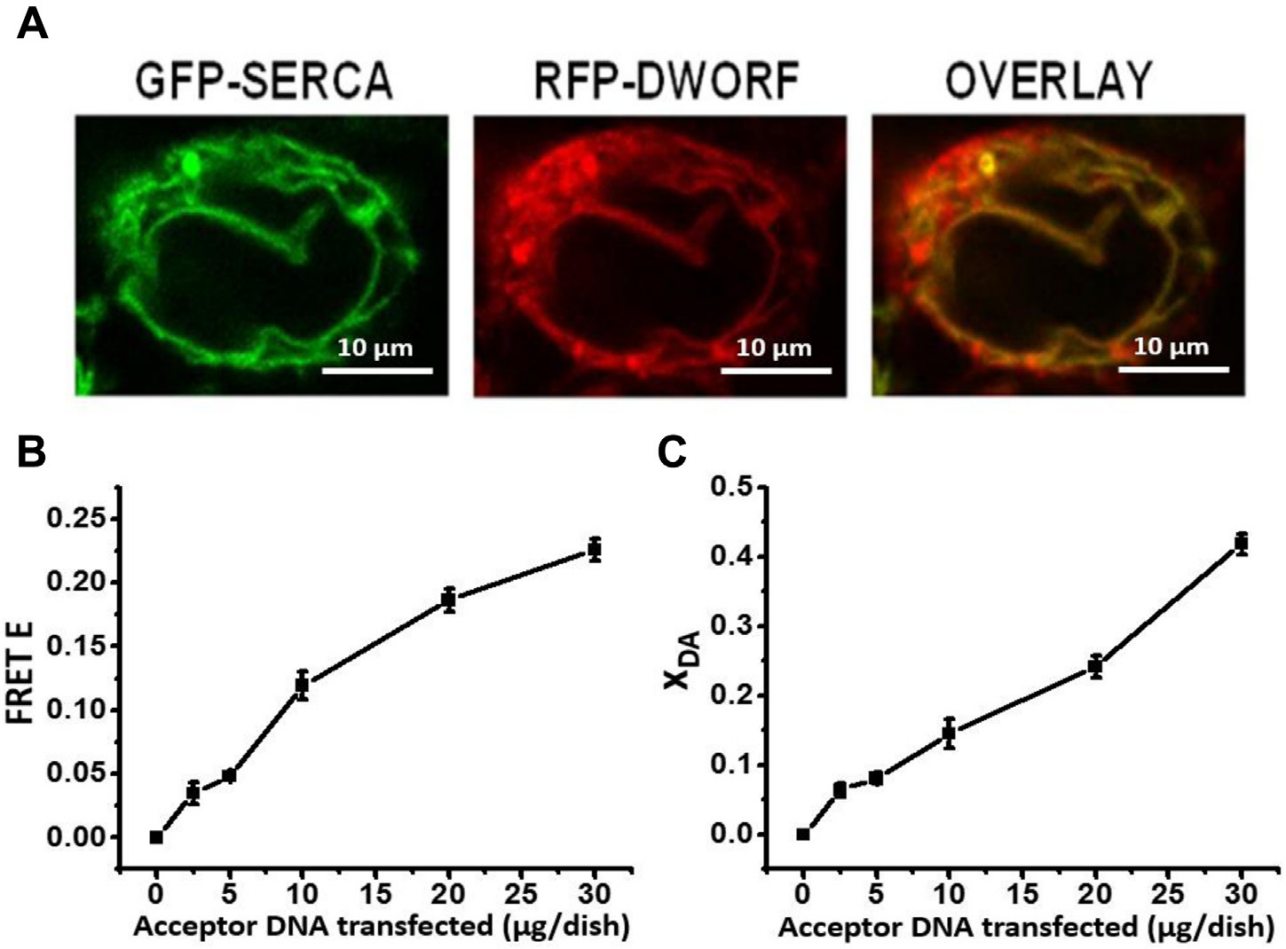
**RFP–DWORF binding to GFP–SERCA2a in live cells.***A*, confocal microscopy images of GFP-SERCA (donor) and RFP–DWORF (acceptor) fluorescence signals. *B*, FRET efficiency E (1 − τ_D+A_/τ_D_) *versus* μg of acceptor DNA seeded per dish. *C*, fraction of SERCA2a containing bound DWORF (X_DA_). Error bars indicate SD (n = 3). GFP, green fluorescent protein; RFP, red fluorescent protein.

To study the SERCA2a–DWORF interaction in living cells, we developed a FRET-based biosensor system by fusing green fluorescent protein (GFP) and tagRFP (red fluorescent protein) to the N termini of SERCA2a and DWORF, respectively. In this biosensor, GFP-SERCA (the FRET donor) is stably expressed, while RFP–DWORF (the FRET acceptor) is transiently expressed to allow varying the expression level. Fluorescence microscopy confirmed that both GFP–SERCA2a and RFP–DWORF are expressed and colocalized in the ER (Fig. 1*A*). In a control experiment, we show that transient transfection of unlabeled DWORF or mutant DWORF into a stable cell line expressing GFP–SERCA2a does not alter the FLT of the donor (Fig. S1). The FRET efficiency (E) and the fraction of donor molecules in proximity to acceptor molecules (X_DA_) were calculated using multiexponential FLT data analysis (see Methods). Due to the R^−6^ dependence of FRET on the donor–acceptor distance R, X_DA_ is a reliable measurement of the fraction of donor-labeled SERCA population that has bound acceptor-labeled PLB or DWORF (16). We found that increasing RFP–DWORF expression results in increasing FRET (E) and X_DA_ (Fig. 1, *B* and *C*), indicating that RFP–DWORF and GFP–SERCA2a are expressed and bound to each other in live cells. FRET values did approach saturation, because the transfection system did not tolerate higher levels of RFP–DWORF DNA.

### DWORF competes with PLB for binding to SERCA2a

**Figure 2:**
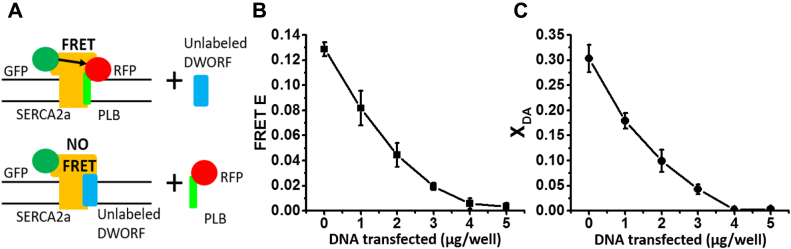
**DWORF competes with PLB for SERCA binding.***A*, schematic illustration of the tested hypothesis, of competition between unlabeled DWORF and RFP-PLB for binding to GFP-SERCA2a. *B*, FRET efficiency E *versus* μg/well of DWORF DNA transfected into 6-well plates stably expressing GFP-SERCA2a and RFP-PLB. *C*, mole fraction (X_DA_) of GFP-SERCA (donor) bound to RFP-PLB (acceptor), for the same samples as in *B*. Error bars indicate SD (n = 3). GFP, green fluorescent protein; RFP, red fluorescent protein.

We transfected increasing amounts of a plasmid carrying unlabeled DWORF into a stable cell line coexpressing GFP-SERCA2a (donor) and RFP-PLB (acceptor) (Fig. 2*A*). In the absence of DWORF, FRET efficiency between labeled SERCA and PLB was E = 0.13 ± 0.01, and the mole fraction of SERCA-bound donor in contact with PLB-bound acceptor was X_DA_ = 0.30 ± 0.03 (Fig. 2, *B* and *C*). Addition of increasing levels of the unlabeled DWORF plasmid resulted in gradually decreased FRET efficiency, reaching a residual level of E = 0.01 ± 0.01 at 5 μg of untagged DWORF DNA per well. This decrease in the interaction between GFP-SERCA2a and RFP-PLB is consistent with the observed substantial decrease in the mole fraction X_DA_ of GFP-SERCA bound to RFP-PLB resolved by multiexponential analysis of the FLT-FRET waveforms (Fig. 2*C*). We conclude that DWORF competes effectively with PLB for binding to SERCA2a.

### TM domain of DWORF is sufficient for functional interaction with SERCA2a

**Figure 3:**
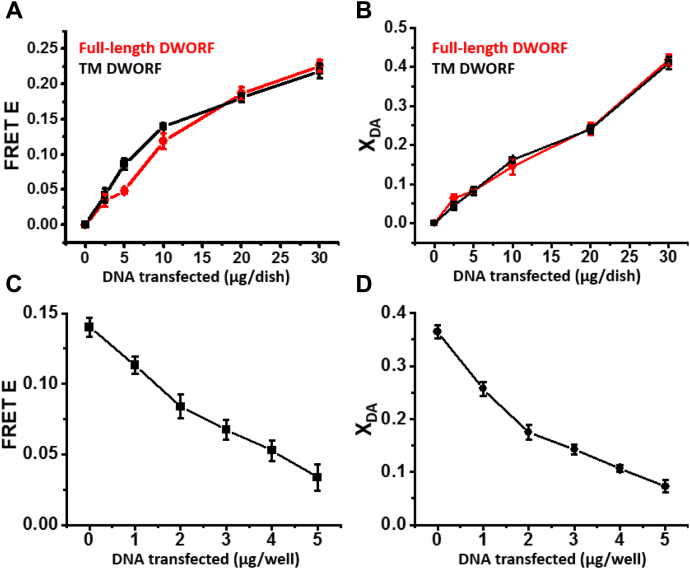
**FRET-detected interaction of SERCA with the isolated TM domain of DWORF is similar to that with full-length DWORF.***A*, FRET efficiency E in HEK293 cells stably expressing GFP–SERCA2a, *versus* μg/dish of DNA transiently transfected into HEK293 cells expressing RFP-TM–DWORF (*black*) or full-length RFP–DWORF (*red*). *B*, fraction X_DA_ of donors (GFP) transferring energy (bound) to acceptor (RFP) determined for samples in panel *A*. *C*, FRET efficiency E for increasing amounts of TM–DWORF DNA transfected into a stable cell line expressing GFP–SERCA2a and RFP–PLB. *D*, fraction X_DA_ of donors transferring energy (bound) to acceptors determined for samples in *C*. Error bars indicate SD (n = 3). GFP, green fluorescent protein; RFP, red fluorescent protein.

DWORF consists of a 23-residue TM domain and an 11-residue cytoplasmic domain that forms a short helix on the cytoplasmic side of the SR membrane (9, 19). To determine which of these domains is responsible for functional interaction with SERCA2a, we engineered a truncated DWORF mutant containing only the TM domain. The RFP-labeled TM domain of DWORF (RFP–TM–DWORF) was transiently transfected into HEK293 cells stably expressing GFP-SERCA2a. With increasing RFP–TM–DWORF expression, we observed increases in both FRET (E) and X_DA_ values, similar to those observed with full-length DWORF (Fig. 3, *A* and *B*). This suggests that the TM domain of DWORF interacts with SERCA similarly to full-length DWORF.

Next, we used an unlabeled TM–DWORF construct, to quantify competitive binding of TM–DWORF to GFP-SERCA2a in the presence of RFP-PLB. As in experiments using unlabeled full-length DWORF (Fig. 2*C*), we observed a decrease in the FRET efficiency E (Fig. 3*C*), corresponding to a decrease in the mole fraction X_DA_ of GFP-SERCA2a transferring energy to RFP-PLB (Fig. 3*D*). We conclude that the TM domain of DWORF is sufficient to compete with PLB for binding to SERCA2a.

### P15 and W22 residues in DWORF are essential for full extent of binding to SERCA2a

**Figure 4:**
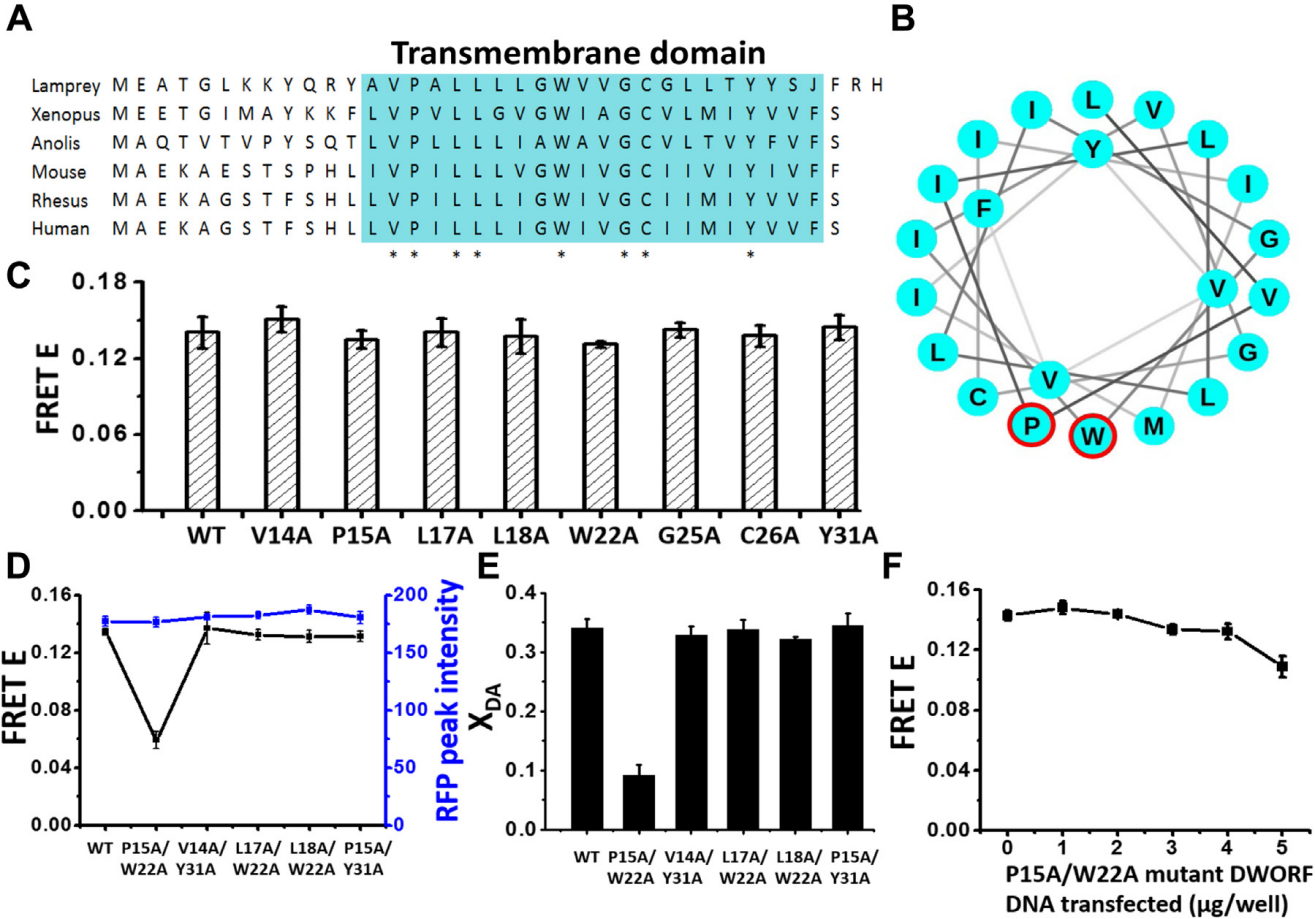
**P15 and W22 residues of DWORF (human isoform) are essential for the full extent of DWORF–SERCA2a complex formation.***A*, DWORF amino acid sequence alignment (19). *Blue region* denotes the putative TM domain. ∗ indicates a residue conserved among all species shown. *B*, helical wheel of DWORF TM domain, with P15 and W22 indicated by *red circles*. *C*, FRET efficiency of RFP–DWORF single-mutant constructs expressed with GFP-SERCA2a. In total, 5 μg/well of each mutant DWORF DNA was transfected into a stable cell line expressing GFP–SERCA2a. *D*, FRET efficiency (*black*; FLT measurement excited at 481 nm) and RFP fluorescence intensity (*blue*; excited at 532 nm) of RFP–DWORF double-mutant constructs expressed under similar conditions. In total, 5 μg/well of each double-mutant DWORF DNA was transfected into a stable cell line expressing GFP–SERCA2a. *E*, fraction X_DA_ of donors transferring energy (bound) to acceptor determined for samples in *D*. *F*, FRET efficiency E of RFP-PLB to GFP-SERCA2a *versus* μg/well DNA of untagged-DWORF (P15A/W22A) transfected. Error bars indicate SD (n = 3). GFP, green fluorescent protein; RFP, red fluorescent protein.

To determine which residues within the TM domain of DWORF are important for complex formation with SERCA2a, DWORF mutants were selected for testing based on evolutionary conservation (Fig. 4*A*). Initially, we tested the effects on single mutations on the ability of fluorescently labeled DWORF constructs to bind to labeled SERCA2a and participate in FRET. However, we did not observe significant differences in the binding affinities of single-mutation DWORF constructs for SERCA2a, as compared with the WT–DWORF controls (Fig. 4*C*). Given that the protein–protein interface between TM domains of the SERCA–DWORF complex may involve interactions involving multiple DWORF residues, we set out to test whether a combination of two mutations is sufficient to ablate complex formation. We found that one combination, a double mutant in which P15 and W22 are both substituted with alanine, showed significant decreases in both FRET efficiency and X_DA_ (Fig. 4, *D* and *E*), indicating that this mutant DWORF binds SERCA2a with a significantly lower affinity than the WT–DWORF controls. The RFP fluorescence intensity was indistinguishable for WT–DWORF and mutants, indicating that their expression levels were similar (Fig. 4*D*). We also tested whether the unlabeled P15A/W22A DWORF mutant can effectively compete for binding to GFP–SERCA in the presence of RFP-PLB and found that competitive binding is substantially decreased by the double mutation (Fig. 4*F*), which is strikingly different from the equivalent measurements above with WT–DWORF (Fig. 2*B*) and TM–DWORF (Fig. 3*C*). Based on the DWORF TM domain sequence, which is predicted to form a single helix, both P15 and W22 residues are expected to be on the same face of the helix (Fig. 4*B*), suggesting that this is the protein–protein interface between the TM domains of SERCA2a and DWORF.

### DWORF activates SERCA2a in the absence of PLB

**Figure 5:**
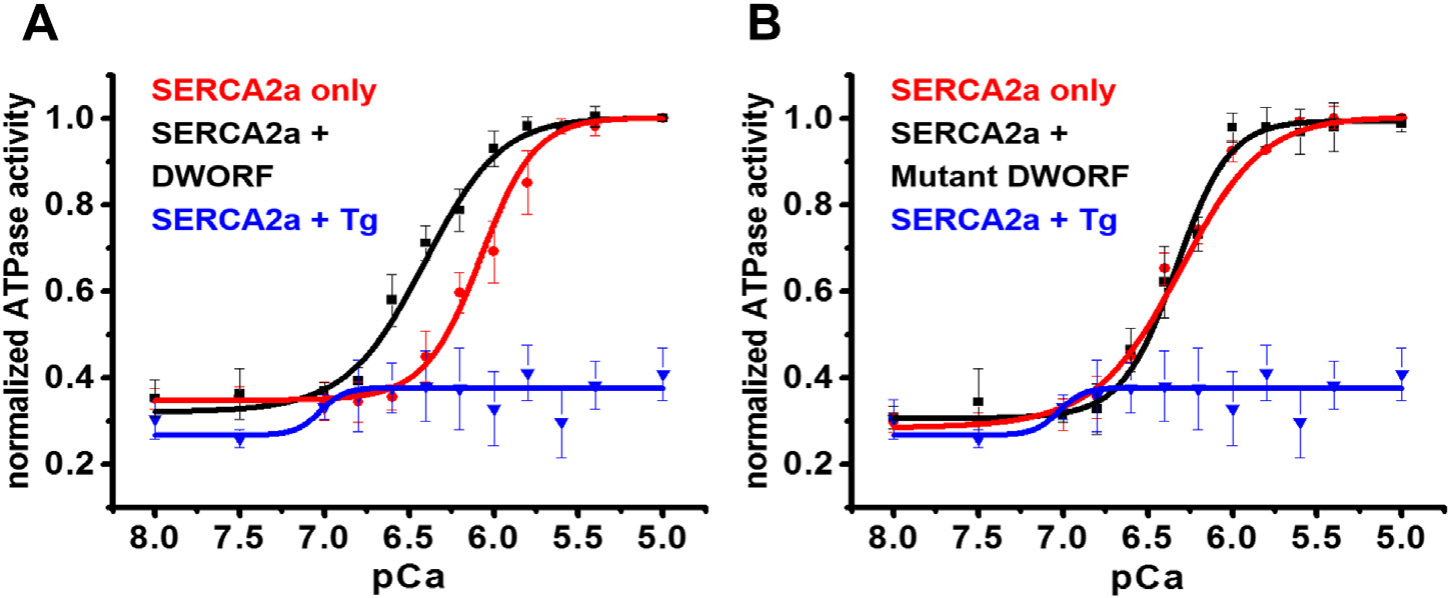
**DWORF activates SERCA2a in the absence of PLB.***A*, Ca-ATPase activity measured in homogenates of cells expressing GFP-SERCA2a and unlabeled DWORF (*black*, pK_Ca_ = 6.36 ± 0.04, Hill n = 0.82 ± 0.02), GFP-SERCA2a only (*red*, pK_Ca_ = 6.08 ± 0.03, Hill n = 1.26 ± 0.03), or GFP–SERCA2a pretreated with 100 nM thapsigargin (Tg) (*blue*). *B*, Ca-ATPase activity measured in homogenates of cells expressing GFP-SERCA2a and unlabeled DWORF–P15/W22 (*black*, pK_Ca_ = 6.34 ± 0.04, Hill n = 1.28 ± 0.01), GFP-SERCA2a only (*red*, pK_Ca_ = 6.32 ± 0.04, Hill n = 1.13 ± 0.02), or GFP-SERCA2a pretreated with 100 nM thapsigargin (Tg) (*blue*). GFP-SERCA2a was stably expressed in HEK293 cells, then 20 μg of DNA per dish (for unlabeled DWORF or unlabeled mutant DWORF) was transiently transfected into the cells. Ca-ATPase assays were performed using cell homogenates, as described in Experimental procedures. Data were fitted using V = V_0_ + V_max_/(1 + 10^−n[pKCa − pCa]^), to determine pK_Ca_, the pCa value for half-maximal activation by Ca. PLB causes a decrease in pK_Ca_ (increase in [Ca^2+^] required for SERCA activation), so activation of SERCA corresponds to an increase in pK_Ca_. Activity was normalized to the control (SERCA2a only), since DWORF had no significant effect on V_max._ Error bars indicate SD (n = 3). GFP, green fluorescent protein.

Based on the FRET measurements above, it is clear that DWORF competes effectively with PLB for binding to SERCA2a (Fig. 2), and this competition has been shown to reverse PLB inhibition of SERCA2a (19). Our FRET measurements show that DWORF also binds to SERCA in the absence of PLB (Fig. 1). To test the hypothesis that DWORF activates SERCA2a directly in the absence of PLB, we performed Ca-ATPase assays in HEK293 cells overexpressing GFP-SERCA2a and varying amounts of DWORF, in the absence of PLB (Fig. 5). Surprisingly, we found that *DWORF activates SERCA2a directly* at subsaturating [Ca^2+^], increasing the apparent Ca affinity of SERCA2a (Fig. 5*A*). Similar results were obtained with RFP–DWORF (Fig. S2), indicating that the tag does not significantly affect DWORF function. As a control, we tested the effects of the double-mutant P15A/W22A DWORF, which displays low affinity toward SERCA2a in our FRET assays, and found that this construct has a substantially decreased effect on Ca-ATPase function (Fig. 5*B*). Since the Ca-ATPase measurements were done using whole cell extracts from transfected cells, and plasma membranes from virtually all cells are known to express relatively high levels of Ca-sensitive nonspecific nucleotidase activity and NADH oxidase activity, not all Ca-ATPase activity measured on homogenized cells is due to SERCA. However, treatment with the SERCA-specific inhibitor thapsigargin (Tg) eliminates most Ca-ATPase activity of the GFP-SERCA2a stable cell line (Fig. 5, *A* and *B*).

## Discussion

By combining structural (FRET) and functional (Ca-ATPase) measurements, the present study reveals a new role for DWORF in calcium regulation. In our model, DWORF binds to SERCA2a, displacing PLB, and activates SERCA *via* two distinct mechanisms: (a) DWORF displaces the inhibitory PLB and (b) DWORF activates SERCA directly, above the level of SERCA in the absence of both DWORF and PLB. Residues P15 and W22 in the TM domain of DWORF are required for DWORF–SERCA binding.

The causes of HF are complex, but growing evidence indicates that central factors include increased basal cytosolic [Ca^2+^], correlated with decreased [Ca^2+^] in the SR lumen. Since SERCA2a is a major determinant of cardiomyocytes cytosolic [Ca^2+^], increasing SERCA2a activity is a potential therapy for a myriad of chronic cardiac pathologies (20). In support of this hypothesis, DWORF enhances SERCA2a activity and contractility in a HF mouse model (21). Here we study this mechanism through direct detection of protein–protein complex formation, using FRET biosensors based on DWORF, PLB, and SERCA2a. We observed that DWORF binds directly to SERCA2a (Fig. 1) and competes effectively with PLB for SERCA2a binding (Fig. 2). This is consistent with previous *in vivo* observations (19) and fluorescence-based measurements showing that SERCA has a higher apparent affinity for DWORF than for PLB (21). DWORF is reported to interact with SERCA2a to form a heterodimer (22). We observed that DWORF activates SERCA2a directly, in the absence of PLB, by enhancing the Ca-ATPase apparent calcium affinity (Fig. 5*A*). This *direct activation of SERCA2a by DWORF* was not detected in previous studies (22). This difference may be due to differences in SERCA2a–DWORF stoichiometry, cell lines used, and/or the presence of protein tags. Given that we measured Ca-ATPase activity using an unlabeled construct at the same SERCA2a–DWORF stoichiometry as in our structure-based assays, we propose that these findings reveal a new role for DWORF, in which DWORF functions as a *bona fide* activator of SERCA2a.

We have shown that the TM domain of DWORF is sufficient for complex formation with SERCA2a and subsequent activation (Fig. 3) (Fig. S3). We identified two resides, P15 and W22, that are important for this interaction (Fig. 4).When P15 and W22 are both mutated to A, the fraction of SERCA2a containing bound DWORF decreases by a factor of 4 (Fig. 5*B*), indicating that this double mutant fails to activate SERCA2a primarily due to decreased binding. However, the double mutant did not completely eliminate FRET or X_DA_, the fraction of SERCA containing bound DWORF (Fig. 4, *D* and *E*), indicating that the double mutation eliminates activation by bound DWORF. Additional DWORF mutants should be tested in the future to determine whether other residues are important for SERCA2a–DWORF interactions. Future structural studies (*e.g.*, crystallography and cryo-EM) will be needed to further explore the mechanism of DWORF’s effect on SERCA2a.

## Conclusion

The current study demonstrates that DWORF binds to SERCA2a, competing with PLB (Fig. 2). The binding of DWORF to SERCA2a depends entirely on the TM domain of DWORF (Fig. 3), and mutation of TM residues P15 and W22 to A greatly decreases the binding of DWORF to SERCA2a (Fig. 4). These results are consistent with the previous proposal that DWORF activates SERCA2a by displacing the inhibitory PLB (19). However, we find that ***DWORF also activates SERCA2a directly*** in the absence of PLB, while the double mutant has no effect (Fig. 5). These results will help to inform future efforts in therapeutic design; a better understanding of the regulation of SERCA2a by small TM proteins is essential for development of new classes of therapeutics in heart failure research.

## Experimental procedures

### Molecular biology

The EGFP (hereafter “GFP”) gene was fused to that of the N terminus of human SERCA2a gene plus a 5-residue linker, as described previously (17, 23). The tagRFP (hereafter “RFP”) gene was similarly fused to the N terminus of the human DWORF or PLB gene. The GFP-SERCA2a construct was cut and pasted with NheI and NotI cutting sites into a pcDNA3.1 vector with ampicillin resistance. The genes for RFP–DWORF, RFP–TM domain of DWORF (RFP–TMDWORF), RFP–PLB, RFP–DWORF, and unlabeled DWORF were separately cut and pasted with NheI and NotI into a pEGFPc1 vector with Kanamycin resistance. RFP–TMDWORF fragment with NheI and NotI cutting site was ordered through the University of Minnesota Genomics Center (UMGC), then inserted into the pEGFPc1 vector. Mutagenesis of DWORF was preformed using a Q5 Site-Directed Mutagenesis Kit (New England BioLabs).

### Cell culture and transfections

HEK293 cells were transfected using Lipofectamine 3000 (Thermo Fisher). SERCA2a constructs were expressed using the mammalian expression vector pcDNA3.1 with G418 resistance. DWORF and PLB constructs were expressed using mammalian expression vector peGFPc1 with puromycin resistance. Twenty-four hours before transfection, HEK293 cells were plated at 0.4 million cells per well in 6-well plates (9.6 cm^2^) or 2 million cells per dish in 10-cm dishes (56.7 cm^2^). For FLT measurements of FRET, cells were be harvested at 1 million cells per ml in PBS 48 h after transfection. To select the stable cell line, 2 days after transfection, 2.0 mg/ml puromycin or 500 μg/ml G418 antibiotic was added to the growth medium. Seven days after antibiotic selection, the remaining cells were enriched by fluorescence-activated cell sorting (FACS). After 3 weeks in culture, there were approximately 100 million cells, generating a stable clone expressing the biosensor at high levels. The stable cell line was maintained using F17 medium (Sigma) + (200 nM/ml) GlutaMAX +2.0 mg/ml puromycin or 500 μg/ml G418.

### Preparation of cell homogenates

Homogenate preparation was performed as previously reported (24). Briefly, cells were centrifuged at 300*g*, washed three times in phosphate buffer solution (PBS, with no magnesium or calcium added, Thermo Scientific), and resuspended in homogenization buffer (0.5 mM MgCl_2_, 10 mM Tris-HCL pH 7.5, DNase I, and protease inhibitor) at 10 million cells/ml. Cells were then incubated on ice for 10 min and broken with the Tissumizer (Tekmar SDT-1810) with three 30-s bursts. After each burst, a 5 min incubation on ice was performed. Homogenization was confirmed with a microscope. After the homogenization, 2× sucrose buffer (1 mM MOPS, 500 mM sucrose, and protease inhibitor) was added for a final cell concentration of 2 mg total protein per ml.

### NADH-enzyme-coupled ATPase activity assay

Functional assays were performed using homogenate preparations expressing either the SERCA2a–DWORF biosensor or the appropriate donor-only control. As previously described (24), an enzyme-coupled, NADH-linked ATPase assay was used to measure SERCA2a Ca-ATPase activity in 96-well microplates. Each well contained assay mix (50 mM MOPS pH 7.0, 100 mM KCl, 5 mM MgCl_2_, 1 mM EGTA, 0.2 mM NADH, 1 mM phosphoenol pyruvate, 10 IU/ml of pyruvate kinase, 10 IU/ml of lactate dehydrogenase, 1 μM of the calcium ionophore A23187 from Sigma), and CaCl_2_ was added to set the free [Ca^2+^] to the desired values. Before starting the assay, 4 mg/ml of cell homogenate, CaCl_2_, and assay mix with or without 100 nM Thapsigargin (Tg) were incubated for 20 min. The assay was then started by adding ATP to a final concentration of 5 mM (200 ml total assay volume), and absorbance was measured at 340 nm in a SpectraMax Plus microplate spectrophotometer (Molecular Devices). Data points were fitted with V = V_0_ + V_max_/(1 + 10^−n[pKCa − pCa]^), to determine pK_Ca_, the pCa value for half-maximal activation by Ca. PLB causes a decrease in pK_Ca_ (increase in [Ca^2+^] required for SERCA activation), and activation of SERCA corresponds to an increase in pK_Ca_.

### FLT measurement and data analysis

FLT measurements were performed by time-correlated single photon counting (TCSPC), as previously described (25). HEK293 cells expressing fluorescent biosensors were harvested at 1 million cells per ml in PBS. TCSPC experiments were performed on samples containing buffer (instrument response function, IRF), untransfected HEK293 (background), and HEK293 expressing donor-only (GFP-SERCA2a) and donor plus acceptor (RFP–DWORF or RFP–PLB). TCSPC experiments were performed using excitation at 481 nm using a subnanosecond pulsed diode laser (LDH-P-C-485, PicoQuant), selecting emitted light using a 540 ± 10 nm bandpass filter (Semrock), and detecting with a PMH-100 photomultiplier (Becker-Hickl).

The FLT-FRET data were analyzed as described previously (13, 25, 26, 27). Two-exponential fits were sufficient for the donor-only and donor–acceptor FLT waveforms, yielding the amplitude-weighted average lifetimes τ_D_ and τ_D+A_, respectively (summarized in Table S1 for all applicable experiments). These values were used to calculate the FRET efficiency E according to E = 1 − (τ_D+A_/τ_D_). To determine the mole fraction X_DA_ of donor (GFP-SERCA2a) bound to acceptor (RFP-PLB), the data for the D + A sample were further analyzed according to F_D+A_(t) = (1 − X_DA_)F_D_(t) + X_DA_F_DA_(t) (13), where the fluorescence of the donor is F_D_(t) = F_D0_exp(−t/τ_D_) and the fluorescence of the donor–acceptor complex F_DA_(t) = F_DA0_exp(−t/τ_DA_).

### Statistical analysis

Analysis of two-group comparisons was done by Student’s *t*-test (∗*p* < 0.05). Data are presented as mean ± standard deviation (SD), and all statistical values were calculated from a minimum of three separate experiments (n = 3).

## Data availability

All data discussed are presented within the article.

## Supporting information

This article contains supporting information.

## Conflict of interest

D. D. T. and R. L. C. hold equity in, and serve as executive officers of, Photonic Pharma LLC. These relationships have been reviewed and managed by the University of Minnesota in accordance with its conflict-of-interest policies. Photonic Pharma had no role in this study. The authors declare no conflicts of interest with regard to this article.
