# Supplementary material for: The transmembrane peptide DWORF activates SERCA2a via dual mechanisms
Source: J Biol Chem. 2021 Feb 11;296:100412. doi: 10.1016/j.jbc.2021.100412 (PMC7988493; doi:10.1016/j.jbc.2021.100412)
Supplement: Supplemental Figures S1–S3 and Table S1 [file mmc1.pdf]

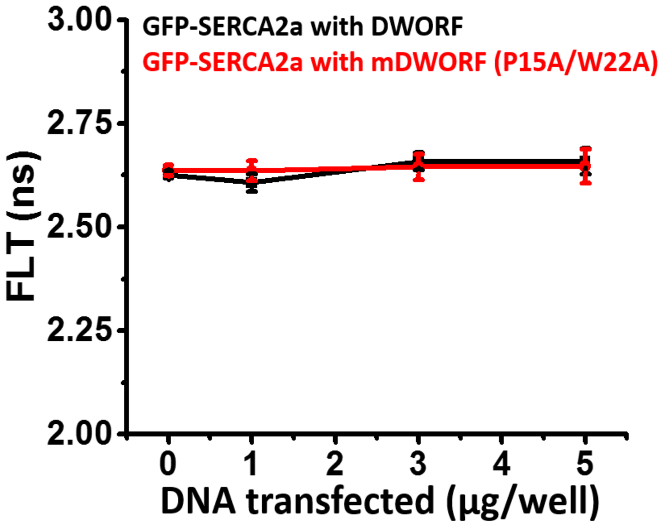

Figure. S1. Unlabeled DWORF does not alter the fluorescence lifetime of GFP-SERCA2a. Transient transfections of unlabeled DWORF (black) or unlabeled mutant DWORF (red) into HEK293 cells stably expressing GFP-SERCA2a. 48 hours after transfection, cells were harvested at 10 million per mL in PBS and used for fluorescence lifetime measurements. Error bars indicate SD (n=3).

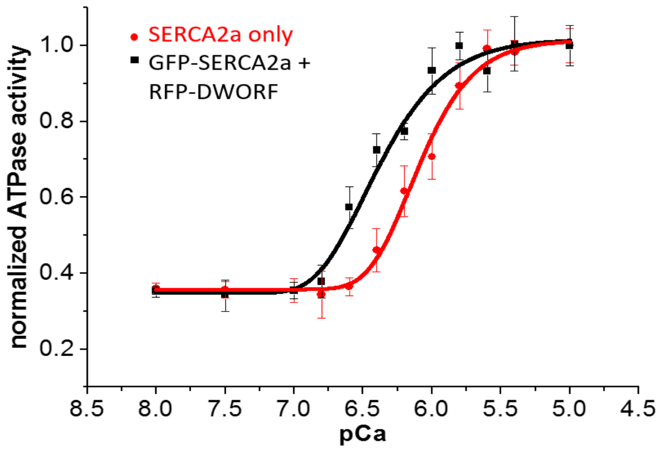

Figure. S2. RFP-DWORF activates SERCA2a directly in the absence of PLB. Ca-ATPase activity measured in homogenates of cells expressing GFP-SERCA2a and RFP-DWORF (black, pKCa = 6.38 ± 0.05, Hill n = 0.92 ± 0.01), or GFP-SERCA2a only (red, pKCa = 6.09 ± 0.03, Hill n = 1.21 ± 0.03). Data points were fitted using $V = V_0 + V_{max}/(1 + 10^{-n[pKCa - pCa]})$, to determine pK_{Ca}, the pCa value for half-maximal activation by Ca. PLB causes a decrease in pK_{Ca} (increase in $[Ca^{2+}]$ required for SERCA activation), and activation of SERCA corresponds to an increase in pK_{Ca}. Activity was normalized to the control (SERCA2a only), since DWORF had no significant effect on V_{max}. Error bars indicate SD (n=3).

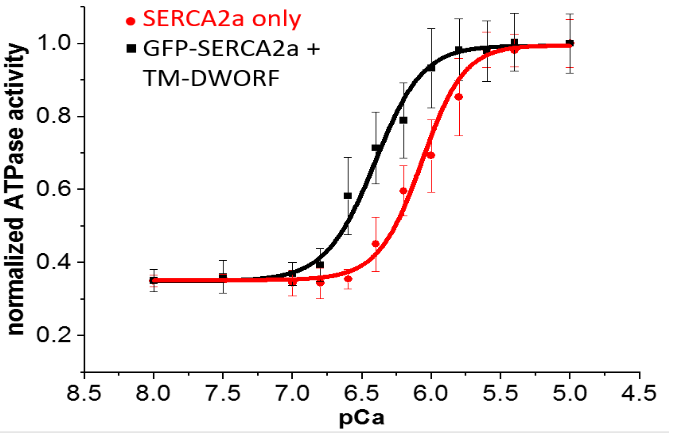

Figure. S3. TM-DWORF can activate SERCA2a directly in the absence of PLB. Ca-ATPase activity measured in homogenates of cells expressing GFP-SERCA2a and RFP-DWORF (black, pKCa = 6.41 ± 0.03, Hill n = 1.09 ± 0.05), or GFP-SERCA2a only (red, pKCa = 6.07 ± 0.03, Hill n = 1.31 ± 0.02). Data points were fitted using $V = V_0 + V_{max}/(1 + 10^{-n[pKCa - pCa]})$, to determine pK_{Ca}, the pCa value for half-maximal activation by Ca. PLB causes a decrease in pK_{Ca} (increase in $[Ca^{2+}]$ required for SERCA activation), and activation of SERCA corresponds to an increase in pK_{Ca}. Activity was normalized to the control (SERCA2a only), since DWORF had no significant effect on $V_{max.}$ Error bars indicate SD (n=3).

Table S1: Fluorescence lifetimes used to calculate FRET E

Fig. 1	lifetime (ns)	Fig. 2	lifetime (ns)	Fig. 3	lifetime (ns)	Fig. 4 C	lifetime (ns)	Fig. 4 D, F	lifetime (ns)
2.5 µg	2.54 ± 0.02	0 µg	2.26 ± 0.03	2.5 µg	2.59 ± 0.01	Wild type	2.25 ± 0.03	Wild type	2.27 ± 0.01
5 µg	2.51 ± 0.01	1 µg	2.40 ± 0.03	5 µg	2.56 ± 0.01	V14A	2.23 ± 0.02	P15A/ W22A	2.47 ± 0.01
10 µg	2.32 ± 0.03	2 µg	2.48 ± 0.02	10 µg	2.50 ± 0.02	P15A	2.27 ± 0.01	V14A/ Y31A	2.26 ± 0.02
20 µg	2.14 ± 0.02	3 µg	2.52 ± 0.02	20 µg	2.40 ± 0.04	L17A	2.25 ± 0.02	L17A/ W22A	2.28 ± 0.03
30 µg	2.04 ± 0.02	4 µg	2.59 ± 0.01	30 µg	2.28 ± 0.01	L18A	2.26 ± 0.03	L18A/ W22A	2.28 ± 0.02
Donor only	2.63 ± 0.01	5 µg	2.60 ± 0.01	Donor only	2.61 ± 0.01	W22A	2.28 ± 0.01	P15A/ Y31A	2.28 ± 0.01
		Donor only	2.63 ± 0.01			G25A	2.25 ± 0.01	Donor only	2.62 ± 0.02
				0 µg	2.24 ± 0.02	C26A	2.26 ± 0.01	0 µg	2.24 ± 0.01
				1 µg	2.33 ± 0.03	Y31A	2.24 ± 0.02	1 µg	2.23 ± 0.02
				2 µg	2.42 ± 0.02	Donor only	2.63 ± 0.01	2 µg	2.24 ± 0.01
				3 µg	2.46 ± 0.03			3 µg	2.27 ± 0.01
				4 µg	2.49 ± 0.03			4 µg	2.27 ± 0.02
				5 µg	2.53 ± 0.03			5 µg	2.33 ± 0.01
				Donor only	2.64 ± 0.01			Donor only	2.62 ± 0.01
